# Factors Associated With Systemic Lupus International Collaborating Clinics/American College of Rheumatology (SLICC/ACR) Damage Index-Defined Organ Damage in Patients with Systemic Lupus Erythematosus: A Cross-Sectional Study From Bangladesh

**DOI:** 10.7759/cureus.117707

**Published:** 2026-10-09

**Authors:** Shadab Saud Sunny, Mohammad Abul Kalam Azad, Md Nazrul Islam

**Affiliations:** 1 Medicine/Rheumatology, Cumilla Medical College and Hospital, Cumilla, BGD; 2 Rheumatology, Bangladesh Medical University, Dhaka, BGD; 3 Rheumatology, Bangabandhu Sheikh Mujib Medical University, Dhaka, BGD

**Keywords:** anemia, antiphospholipid antibodies, bangladesh, cross-sectional study, damage accumulation, disease activity, organ damage, slicc/acr damage index, systemic lupus erythematosus, vasculitis

## Abstract

Introduction

Systemic lupus erythematosus (SLE) is a chronic autoimmune disease with a wide range of clinical manifestations and an unpredictable course. Although disease activity can fluctuate over time, some organ injury becomes permanent and contributes substantially to the long-term burden of SLE. The Systemic Lupus International Collaborating Clinics/American College of Rheumatology Damage Index (SLICC/ACR DI or SDI) provides a standardized way to capture such irreversible damage. Data describing the burden and clinical correlates of SDI-defined damage in Bangladesh remain limited. This study therefore examined the pattern of established organ damage and the clinical and laboratory factors associated with it among Bangladeshi patients with SLE.

Methodology

This single-center, cross-sectional observational study included 168 consecutive patients with SLE who fulfilled the 2019 European League Against Rheumatism/American College of Rheumatology classification criteria. Irreversible damage was assessed using the SDI, with a score of ≥1 defining established damage. Demographic, clinical, disease activity, and laboratory characteristics were compared between patients with and without established damage. Multivariable logistic regression was used to identify factors independently associated with damage. A sensitivity analysis excluded patients with a disease duration of <1 year. Because antiphospholipid antibody (aPL) results were available for only 48 participants, aPL status was examined separately in an exploratory nested analysis.

Results

We identified established organ damage in 51 (30.4%) patients. Among those with damage, 39 (76.5%) had an SDI score of 1. Skin involvement was the most frequently recorded damage domain, affecting 30 (17.9%) patients and 30 (58.8%) of those with established damage. In the primary multivariable model, longer disease duration (adjusted odds ratio (aOR) 1.11 per year, 95% confidence interval (CI) 1.02-1.20; *P* = 0.019), anemia (aOR 2.58, 95% CI 1.22-5.46; *P* = 0.013), and vasculitis (aOR 4.68, 95% CI 1.70-12.91; *P* = 0.003) were independently associated with established damage. The Safety of Estrogens in Lupus Erythematosus National Assessment-Systemic Lupus Erythematosus Disease Activity Index (SELENA-SLEDAI) was not independently associated with damage after adjustment (aOR 1.05 per point, 95% CI 0.99-1.11; *P* = 0.086). After excluding 20 patients with a disease duration of <1 year, anemia and vasculitis remained associated with damage, whereas disease duration and SELENA-SLEDAI did not. In the exploratory analysis of 48 patients with available aPL results, aPL positivity was associated with established damage (aOR 5.95, 95% CI 1.44-24.55; *P* = 0.014), but the small sample and incomplete testing limit the strength of this finding.

Conclusions

Established SDI-defined organ damage was present in approximately one-third of patients in this Bangladeshi SLE population, with skin damage being the most common domain. Anemia and vasculitis showed the most consistent associations with established damage, including after exclusion of patients with very early disease. Because the study was cross-sectional, these findings represent associations rather than causal relationships. The exploratory association between aPL positivity and damage should be tested in larger studies with systematic antibody assessment.

## Introduction

Systemic lupus erythematosus (SLE) is a chronic autoimmune disease with a wide range of symptoms and a relapsing-remitting course. The 2019 European League Against Rheumatism/American College of Rheumatology (EULAR/ACR) classification criteria provide a reliable way to classify systemic lupus erythematosus (SLE) [1]. Despite advances in diagnosis and treatment, many patients still suffer permanent organ damage, which leads to significant health problems and increased risk of death. The Systemic Lupus International Collaborating Clinics/American College of Rheumatology (SLICC/ACR) Damage Index (SDI) was developed to measure this irreversible damage across various organs since the onset of SLE. It has been validated and used worldwide [2].

The development of organ damage in SLE is multifactorial. Cumulative inflammatory disease activity, vascular and thrombotic complications, renal involvement, treatment-related toxicity, and comorbid conditions may all contribute to irreversible damage. Evidence from systematic reviews and longitudinal cohorts demonstrates a consistent relationship between accumulated organ damage and adverse outcomes, including mortality [3].

Several longitudinal studies have identified factors associated with damage accrual. Analyses from the SLICC inception cohort and Hopkins Lupus Cohort have implicated disease duration, disease activity, glucocorticoid exposure, hypertension, age, and other disease-related factors [4,5]. Data from the LUMINA cohort have also highlighted anemia in relation to disease activity and subsequent damage [6].

The pattern of damage may differ between populations. A systematic review of the Asia-Pacific region identified older age, glucocorticoid exposure, longer disease duration, and disease activity among factors associated with damage, while lupus nephritis was particularly relevant to renal and overall damage. Hydroxychloroquine use was associated with lower overall damage [7]. SLE has also been reported to be relatively common and to involve more renal and central nervous system (CNS) damage, as well as higher rates of cyclophosphamide use, among Asian populations compared with some Western populations [8]. Studies from southern China have demonstrated that major flares and the use of cyclophosphamide were associated with damage accrual among patients with SLE [9], while a large Korean cohort of 996 patients evaluated clinical manifestations, disease activity, and organ damage in comparison with other Asian populations and reported that musculoskeletal, renal, and neuropsychiatric damage was prevalent [10]. Similarly, a cohort study from Malaysia has documented age, Indian ethnicity, low C3 level, antiphospholipid syndrome (APS), and neuropsychiatric systemic lupus erythematosus (NPSLE) as factors associated with damage in patients with multiethnic SLE [11]. Another retrospective Malaysian cohort study observed that neuropsychiatric, skin, and musculoskeletal damage were common. Factors associated with damage were hypertension, exposure to cyclophosphamide, and lower prednisolone dose [12].

Renal involvement appears to be particularly important in determining long-term outcomes. In Pakistan, a retrospective cohort of 198 patients with SLE reported organ damage in about 76% of patients, with renal disease being the commonest cause, and identified renal involvement and infections as independent predictors of mortality [13]. A subsequent report from the same group found early renal damage to be associated with mortality in patients with lupus [14]. A Japanese single-center cross-sectional study has also demonstrated that age, hypertension, serum creatinine levels, and APS were associated with increased damage; administration of bisphosphonate was associated with decreased damage [15]. In a large Chinese inception cohort of newly diagnosed patients, SDI-defined damage was present in 22.4% at baseline and was more frequent in men than in women [16]. Longitudinal data from the Asia Pacific Lupus Collaboration (APLC) cohort have also shown that flares independently increase the risk of subsequent damage accrual [17]. A previous cross-sectional study from the same clinical setting in Bangladesh found comparable cumulative organ damage between male and female patients, although disease activity was higher in men [18]. Furthermore, other longitudinal international data from the APLC cohort indicate that active lupus nephritis, age, and cumulative prednisolone dose were independently associated with irreversible organ damage in patients with SLE; Asian ethnicity was protective against non-renal damage accrual [19]. In a large APLC analysis of 4,102 patients, mucocutaneous damage accrued in only 4.3% of those assessed, most often as alopecia, whereas persistent mucocutaneous disease activity was associated with a higher risk of such damage [20].

Vascular complications are another potential pathway to irreversible injury. Antiphospholipid antibodies (aPLs) have been associated with early and overall damage in patients with SLE, supporting the importance of vascular and thrombotic mechanisms alongside inflammatory disease manifestations [21,22]. Treatment exposure may also influence the balance between disease control and treatment-related harm. Hydroxychloroquine has been associated with less damage accrual, whereas greater cumulative glucocorticoid exposure has been linked to increased damage [23,24]. Importantly, damage can continue to accumulate even when overt clinical or serological disease activity is absent, while sustained remission and low disease activity are associated with lower subsequent damage accrual [25,26].

More recent research has emphasized examining damage by individual organ domains rather than relying only on a total SDI score, because different types of irreversible injury may arise through different clinical pathways [27].

Evidence from Bangladesh remains limited. A previous analysis from the same clinical setting examined factors associated with lupus-associated vasculitis [28]. The present study addresses a different question by focusing on the distribution of SDI-defined irreversible damage and the factors associated with established damage.

The primary objective of this study was to describe the distribution of SLICC/ACR Damage Index-defined organ damage and to identify demographic, clinical, laboratory, and disease-related factors associated with established damage (SDI ≥1) among patients with SLE in Bangladesh. The secondary, exploratory objectives were to examine the association of aPL positivity with established damage in the subset of patients with available testing and to explore the association of medication exposure with damage. These analyses were considered exploratory because aPL testing was incomplete and standardized cumulative treatment-exposure data were not consistently available. Given the cross-sectional design, the study can identify associations but cannot establish temporal relationships or causality.

## Materials and methods

Study design and setting

This was a single-center, cross-sectional observational study conducted in the Department of Rheumatology, Bangladesh Medical University (BMU), Dhaka, Bangladesh. The analysis included 168 consecutive patients with SLE who fulfilled the 2019 EULAR/ACR classification criteria [1]. The study population was derived from a previously described Bangladeshi SLE dataset used to investigate lupus vasculitis; the source study was conducted from December 2019 to January 2021 [28]. The present work is a secondary analysis of that dataset with a different primary outcome, outcome definition, and analytical objective. The earlier study focused on lupus vasculitis, whereas the present analysis evaluates cumulative SDI-defined organ damage.

Participants

The analysis included 168 eligible patients from the source dataset. Patients for whom the available clinical information was insufficient to calculate an SDI score, or who had an overlap connective-tissue disease, were considered ineligible. Disease duration was defined as the interval between the diagnosis of SLE and the study assessment. Twenty participants had a disease duration of <1 year. They were retained in the primary analysis and excluded only in the prespecified sensitivity analysis..

Assessment of organ damage

Irreversible organ damage was assessed using the SDI, which records cumulative, non-reversible impairment across 12 organ systems [2]. The primary outcome was established damage, defined as an SDI score ≥1; an SDI score of 0 was considered no established damage. SDI items were scored according to the original SLICC/ACR methodology. Most items require persistence for at least six months, whereas specified irreversible events are scored according to their individual definitions [2]. SDI scores were obtained from clinical examination and medical-record information available in the source dataset. SDI scoring was performed by a single treating rheumatologist at the time of the source study, with items verified against the medical record. Formal inter-rater reliability data for SDI scoring were not available in the source dataset. Because a patient could have damage in more than one organ system, domain-specific frequencies were not mutually exclusive.

Assessment of disease activity and clinical characteristics

Current disease activity was assessed using the Safety of Estrogens in Lupus Erythematosus National Assessment-Systemic Lupus Erythematosus Disease Activity Index (SELENA-SLEDAI) at a single assessment [29,30]. Demographic and clinical variables included age, sex, body mass index (BMI), disease duration, anemia, lupus nephritis, NPSLE, serositis, antiphospholipid syndrome (APS), deep-vein thrombosis (DVT), and vasculitis. Anemia was defined using World Health Organization hemoglobin criteria. Diagnoses of lupus nephritis were based on persistent proteinuria >0.5 g/day or active urinary sediment, with biopsy confirmation when available; NPSLE was defined according to the ACR 1999 case definitions for NPSLE; serositis was defined according to ACR-EULAR SLE classification criteria; APS was defined according to clinical diagnosis and aPL positivity using the immunological framework applied for SLE classification; and DVT was defined as a clinical diagnosis confirmed by duplex ultrasonography.

Vasculitis was defined using the clinical criteria used in the source study, including palpable purpura, erythematous punctate lesions, tender erythematous macules or papules, tender finger nodules, ulcers, cutaneous nodules, digital gangrene, mesenteric vasculitis, and mononeuritis multiplex. Cutaneous vasculitis was diagnosed clinically and, when performed, supported by a dermatologist-reviewed biopsy. Vasculitic digital gangrene was diagnosed clinically and by duplex ultrasonography, with biopsy findings considered when available. Vasculitis was included in the multivariable model because of its clinical relevance to irreversible tissue injury and its previously reported association with disease burden in this population [28].

Laboratory variables and aPLs

Laboratory variables included erythrocyte sedimentation rate (ESR) and other parameters available in the source dataset. Standardized information on cumulative glucocorticoid exposure (including prednisolone dose and duration) and hypertension was insufficient for inclusion in the primary multivariable model and therefore represents a potential source of residual confounding.

aPL testing was available for 48 of the 168 participants. Patients without documented aPL results were not considered aPL-negative; they remained eligible for the primary analysis and were excluded only from the aPL-specific analysis. aPL positivity was defined using the immunological framework applied for SLE classification, including anticardiolipin antibodies (IgG or IgM) at medium-to-high titer, anti-β2-glycoprotein I antibodies, or a positive lupus anticoagulant test. Because formal APS classification requires persistent laboratory positivity on repeat testing at least 12 weeks apart, a single available aPL measurement was not treated as sufficient to diagnose APS.

Statistical analysis

Analyses were performed using IBM SPSS Statistics version 25 (IBM Corp., Armonk, NY). Continuous variables were summarized as mean ± standard deviation or median with interquartile range (IQR), as appropriate; categorical variables were reported as *n* (%). Participants were divided according to established damage status (SDI ≥ 1 versus SDI = 0). Continuous variables were compared using the Mann-Whitney U test, while categorical variables were compared using Pearson's chi-square test or Fisher's exact test, as appropriate. All tests were two-sided, and *P* < 0.05 was considered statistically significant.

Univariable logistic regression was used to estimate crude odds ratios (ORs) with 95% confidence intervals (CIs) for individual associations with established damage. A multivariable logistic regression model was then fitted using covariates selected from the univariable and bivariate analyses together with clinical relevance, and with the number of covariates limited to preserve approximately 12 outcome events per predictor. Five variables had *P* < 0.05 in the bivariate analyses (disease duration, SELENA-SLEDAI, anemia, leukopenia, and vasculitis); because the 51 outcome events allowed no more than four predictors at this ratio, four were entered. Leukopenia was not entered because it is a component of SELENA-SLEDAI. Adjusted odds ratios (aORs) and 95% CIs are reported. Because of the cross-sectional design, odds ratios were interpreted as measures of association rather than prospective risk estimates or causal effects.

Sensitivity and exploratory analyses

A prespecified sensitivity analysis excluded the 20 participants with a disease duration of <1 year, leaving 148 participants, including 50 with established damage.

Because aPL testing was available for only 48 participants, a separate nested multivariable logistic regression model examined the association between aPL positivity and established damage after adjustment for SELENA-SLEDAI. This analysis was considered exploratory because of the small sample, incomplete testing, limited number of outcome events, and imprecision of the estimates.

Medication exposure was also examined as an exploratory secondary analysis. Hydroxychloroquine, corticosteroids, mycophenolate mofetil, azathioprine, and cyclophosphamide were analyzed separately as binary exposure variables. Cumulative dose, duration, adherence, and standardized historical exposure were not consistently available; therefore, these analyses were not designed to estimate cumulative treatment effects and may be affected by confounding by indication.

Missing data and model assessment

Available data were used for the primary descriptive analyses. Participants without documented aPL results were excluded only from the aPL-specific analysis. Regression models were based on participants with complete data for the variables included in each model. The primary model included 168 participants, 51 damage events, and four predictors; the sensitivity model included 148 participants, 50 damage events, and four predictors; and the exploratory aPL model included 48 participants, 18 damage events, and two predictors. Multicollinearity was assessed using variance inflation factors (all ≤ 1.21) and calibration using the Hosmer-Lemeshow test (primary model *P* = 0.053; sensitivity model *P* = 0.119).

Relationship to the previous publication

The study population overlaps with the population reported previously by Sunny et al. in a study of lupus vasculitis in Bangladesh [28]. This overlap is disclosed to maintain transparency. The previous publication evaluated lupus vasculitis as its primary outcome, whereas the present study examines SDI-defined cumulative organ damage. The current analysis additionally evaluates the distribution of damage domains, factors associated with established damage, a prespecified sensitivity analysis excluding patients with a disease duration of <1 year, and an exploratory aPL analysis. Accordingly, this manuscript represents a secondary analysis addressing a distinct research question rather than a replication of the earlier analysis.

The study was reported in accordance with the Strengthening the Reporting of Observational Studies in Epidemiology (STROBE) recommendations for cross-sectional studies [31].

## Results

Study population and prevalence of established damage

A total of 168 patients with SLE were included in the final analysis. Established SDI-defined damage (SDI ≥1) was present in 51 patients (30.4%), whereas 117 patients (69.6%) had an SDI score of 0.

Twenty participants had a disease duration of <1 year and were retained in the primary analysis. Among the 48 participants with available aPL testing, 23 (47.9%) were aPL-positive.

Distribution and patterns of organ damage

Among the 168 patients with SLE, 51 (30.4%) had established organ damage, defined by an SDI score ≥1, whereas 117 (69.6%) had no documented damage. Most patients with damage had a relatively low cumulative burden: 39 patients (23.2% of the entire study population; 76.5% of those with damage) had an SDI score of 1. Eight patients (4.8%; 15.7% of those with damage) had a score of 2, three (1.8%; 5.9%) had a score of 3, and one patient (0.6%; 2.0%) had a score of 4. Thus, although approximately one-third of the study population had established damage, severe or multisystem damage was uncommon.

Cutaneous damage was the most frequent damage domain, occurring in 30 patients (17.9% of the total SLE study population and 58.8% of those with damage). Alopecia accounted for almost all cutaneous damage, affecting 29 patients (17.3%), while extensive scarring or panniculitis-related damage was recorded in one patient (0.6%). Neuropsychiatric damage was identified in seven patients (4.2% of the study population; 13.7% of those with damage). Within this domain, major psychosis and transverse myelitis were each documented in two patients (1.2%), while cognitive impairment, cerebrovascular accident, cranial neuropathy, and peripheral neuropathy were each recorded in one patient (0.6%).

Musculoskeletal damage was present in five patients (3.0%), including avascular necrosis in three (1.8%), muscle atrophy or weakness in two (1.2%), and deforming arthritis in two (1.2%). Peripheral vascular damage was also documented in five patients (3.0%). This included minor tissue loss in one patient, significant tissue loss in two, and venous thrombosis with swelling, ulceration, or venous stasis in three patients.

Renal damage was present in three patients (1.8%), including a reduced glomerular filtration rate to <50% of normal in two patients (1.2%) and end-stage renal disease in one (0.6%). Pulmonary damage was documented in three patients (1.8%), comprising pulmonary hypertension in three patients and pulmonary fibrosis in one. Ocular damage occurred in three patients (1.8%) and included cataract, central retinal artery occlusion, and optic atrophy. Cardiovascular damage was least frequent, occurring in one patient (0.6%), who had cardiomyopathy.

Overall, the distribution of damage demonstrated a predominantly low SDI burden, with cutaneous manifestations accounting for the largest proportion of established damage. Irreversible involvement of major organs, including the kidneys, lungs, eyes, and cardiovascular system, was less frequent but clinically consequential (Table 1).

**Table 1:** Distribution of SLICC/ACR Damage Index scores and documented organ-damage domains. Domain frequencies are not mutually exclusive because individual patients could have damage in more than one organ system. Percentages are based on *n* = 168 overall and *n* = 51 among patients with established damage. SLICC/ACR, Systemic Lupus International Collaborating Clinics/American College of Rheumatology; SDI, SLICC/ACR Damage Index

Measure/domain	n	% Overall (*n*/168)	% Among patients with damage (*n*/51)
SLICC/ACR Damage Index score			
0	117	69.60%	-
1	39	23.20%	76.47%
2	8	4.80%	15.70%
3	3	1.80%	5.90%
4	1	0.60%	2.00%
Damage domains			
Skin	30	17.90%	58.80%
Alopecia	29	17.30%	56.90%
Extensive scarring or panniculum, other than the scalp and pulp space	1	0.60%	2.00%
Neuropsychiatric	7	4.20%	13.70%
Cognitive impairment (memory deficit)	1	0.60%	2.00%
Major psychosis	2	1.20%	3.90%
Cerebrovascular accident ever	1	0.60%	2.00%
Cranial neuropathy (Bell’s palsy)	1	0.60%	2.00%
Peripheral neuropathy	1	0.60%	2.00%
Transverse myelitis	2	1.20%	3.90%
Musculoskeletal	5	3.00%	9.80%
Muscle atrophy/weakness	2	1.20%	3.90%
Avascular necrosis	3	1.80%	5.90%
Deforming arthritis	2	1.20%	3.90%
Peripheral vascular	5	3.00%	9.80%
Minor tissue loss (pulp space)	1	0.60%	2.00%
Significant tissue loss ever (e.g., loss of digit or limb, resection) (score 2, if > 1)	2	1.20%	3.90%
Venous thrombosis with swelling, ulceration, or venous stasis	3	1.80%	5.90%
Renal	3	1.80%	5.90%
Estimated or measured GFR < 50%	2	1.20%	3.90%
End-stage renal disease (regardless of dialysis or transplantation)	1	0.60%	2.00%
Pulmonary	3	1.80%	5.90%
Pulmonary hypertension	3	1.80%	5.90%
Pulmonary fibrosis	1	0.60%	2.00%
Ocular	3	1.80%	5.90%
Any cataract ever	1	0.60%	2.00%
Retinal change (central retinal artery occlusion)	1	0.60%	2.00%
Optic atrophy	1	0.60%	2.00%
Cardiovascular	1	0.60%	2.00%
Cardiomyopathy	1	0.60%	2.00%

Characteristics associated with established damage

Patients with established damage had a longer median disease duration than those without damage (4.0 (IQR 2.0-9.0) vs. 3.0 (IQR 1.0-6.0) years; *P* = 0.020) and higher median SELENA-SLEDAI scores (11.0 (IQR 5.0-15.0) vs. 6.0 (IQR 2.0-12.0); *P* = 0.001).

Anemia and leukopenia were more frequent among patients with established damage than among those without damage (anemia: 29 (56.9%) vs. 41 (35.0%); *P* = 0.011; leukopenia: 8 (15.7%) vs. 5 (4.3%); *P* = 0.023). Vasculitis was also more frequent among patients with established damage (15 (29.4%) vs. 8 (6.8%); *P* < 0.001).

Age, BMI, ESR, sex, lupus nephritis, NPSLE, serositis, APS, and DVT did not differ significantly between groups (Table 2).

**Table 2:** Comparison of demographic, clinical, and laboratory characteristics with SLICC/ACR Damage Index-defined damage status. Continuous variables are presented as median (IQR) and were compared using the Mann-Whitney U test; categorical variables are presented as *n* (%) and female sex, anemia, lupus nephritis, NPSLE, and vasculitis were compared using Pearson's chi-square test; leukopenia, serositis, and APS were compared using Fisher's exact test. *P < 0.05 was considered statistically significant; significant values are denoted with an asterisk. Exact Mann-Whitney U statistics are reported in the *Test statistic* column. BMI, body mass index; ESR, erythrocyte sedimentation rate; IQR, interquartile range; leukopenia, defined as total leukocyte count less than 3,000/mm^3^; SLICC/ACR, Systemic Lupus International Collaborating Clinics/American College of Rheumatology; APS, antiphospholipid syndrome; SELENA-SLEDAI, Safety of Estrogens in Lupus National Assessment-Systemic Lupus Erythematosus Disease Activity Index

Variable	Damage (*n* = 51)	No damage (*n* = 117)	Test statistic	*P*-value
Age, years, median (IQR)	28.0 (23.0-31.5)	26.0 (20.0-31.0)	2515.5	0.106
Disease duration, years, median (IQR)	4.0 (2.0-9.0)	3.0 (1.0-6.0)	2310.5	0.020*
SELENA-SLEDAI, median (IQR)	11.0 (5.0-15.0)	6.0 (2.0-12.0)	2062	0.001*
BMI, kg/m², median (IQR)	23.0 (20.9-25.6)	22.6 (19.6-26.1)	2854.5	0.658
ESR, mm/hour, median (IQR)	51.0 (26.0-89.5)	37.0 (22.0-58.0)	2417.5	0.051
Female sex, *n* (%)	48 (94.1%)	110 (94.0%)	-	1
Anemia, *n* (%)	29 (56.9%)	41 (35.0%)	-	0.008*
Leukopenia, *n* (%)	8 (15.7%)	5 (4.3%)	-	0.023*
Lymphopenia, *n* (%)	15 (29.4%)	21 (17.9%)	-	0.096
Lupus nephritis, *n* (%)	21 (41.2%)	52 (44.4%)	-	0.737
Neuropsychiatric SLE, *n* (%)	14 (27.5%)	25 (21.4%)	-	0.429
Serositis, *n* (%)	6 (11.8%)	9 (7.7%)	-	0.391
Antiphospholipid syndrome, *n* (%)	7 (13.7%)	7 (6.0%)	-	0.128
Vasculitis, *n* (%)	15 (29.4%)	8 (6.8%)	-	<0.001*

Univariable logistic regression

In univariable logistic regression, higher SELENA-SLEDAI scores (OR 1.07 per point, 95% CI 1.02-1.12; *P* = 0.007), anemia (OR 2.44, 95% CI 1.25-4.78; *P* = 0.009), and vasculitis (OR 5.68, 95% CI 2.22-14.49; *P* < 0.001) were associated with established damage. Age, disease duration, lupus nephritis, NPSLE, APS, and DVT were not significantly associated with established damage (Table 3).

**Table 3:** Univariable logistic regression analysis of factors associated with organ damage defined by the SLICC/ACR Damage Index Outcome: SLICC/ACR Damage Index ≥1. Univariable binary logistic regression using the Wald test was performed to estimate ORs, expressed per one-unit increase for continuous variables and as the odds associated with the presence versus absence of binary variables. *P<0.05 was considered statistically significant; statistically significant values are denoted by an asterisk. SLICC/ACR, Systemic Lupus International Collaborating Clinics/American College of Rheumatology; OR, odds ratio; SELENA-SLEDAI, Safety of Estrogens in Lupus National Assessment-Systemic Lupus Erythematosus Disease Activity Index; CI, confidence interval

Predictor	OR	95% CI	Wald χ²	*P*-value
Age, per year	1.03	0.98-1.07	1.27	0.26
Disease duration, per year	1.06	0.98-1.14	2.28	0.131
SELENA-SLEDAI, per point	1.07	1.02-1.12	7.34	0.007*
Anemia	2.44	1.25-4.78	6.79	0.009*
Lupus nephritis	0.88	0.45-1.70	1.04	0.311
Neuropsychiatric SLE	1.39	0.65-2.97	0.73	0.392
Antiphospholipid syndrome	2.5	0.83-7.54	2.64	0.104
Vasculitis	5.68	2.22-14.49	13.19	<0.001*

Primary multivariable analysis

In the multivariable model, longer disease duration, anemia, and vasculitis were significantly associated with established SDI-defined damage. Each additional year of disease duration was associated with higher odds of established damage (aOR 1.11, 95% CI 1.02-1.20; *P* = 0.019). Anemia was associated with approximately 2.6-fold higher odds of damage (aOR 2.58, 95% CI 1.22-5.46; *P* = 0.013). Vasculitis showed the strongest association with established damage (aOR 4.68, 95% CI 1.70-12.91; *P* = 0.003). SELENA-SLEDAI was not significantly associated with established damage after adjustment for the other covariates (aOR 1.05 per point, 95% CI 0.99-1.11; *P* = 0.086) (Table 4).

**Table 4:** Primary and sensitivity multivariable logistic regression analyses of factors associated with organ damage defined by the SLICC/ACR Damage Index. Outcome: SLICC/ACR Damage Index ≥1. aOR, adjusted odds ratio, from multivariable binary logistic regression using the Wald test; CI, confidence interval. Model 1 included 168 participants, 51 damage events, and four predictors (12.75 events per predictor). Model 2 included 148 participants, 50 damage events, and four predictors (12.5 events per predictor). *P < 0.05 was considered statistically significant; statistically significant values are denoted by an asterisk. SLICC/ACR, Systemic Lupus International Collaborating Clinics/American College of Rheumatology; SELENA-SLEDAI, Safety of Estrogens in Lupus National Assessment–Systemic Lupus Erythematosus Disease Activity Index

Model	Predictor	aOR	95% CI	*P*-value
Model 1, *n* = 168	Disease duration, per year	1.11	1.02-1.20	0.019*
	SELENA-SLEDAI, per point	1.05	0.99-1.11	0.086
	Anemia	2.58	1.22-5.46	0.013*
	Vasculitis	4.68	1.70-12.91	0.003*
Model 2, disease duration ≥1 year, *n *= 148	Disease duration, per year	1.06	0.97-1.16	0.171
	SELENA-SLEDAI, per point	1.04	0.98-1.10	0.225
	Anemia	2.45	1.14-5.26	0.021*
	Vasculitis	5.29	1.77-15.74	0.003*

Sensitivity analysis excluding patients with disease duration <1 year

After excluding 20 patients with disease duration <1 year, 148 participants remained, including 50 (33.8%) with established damage. In the sensitivity model, disease duration was no longer significantly associated with established damage (aOR 1.06 per year, 95% CI 0.97-1.16; *P* = 0.171), nor was SELENA-SLEDAI (aOR 1.04 per point, 95% CI 0.98-1.10; *P* = 0.225). In contrast, anemia remained significantly associated with established damage after adjustment (aOR 2.45, 95% CI 1.14-5.26; *P* = 0.021), as did vasculitis (aOR 5.29, 95% CI 1.77-15.74; *P* = 0.003) (Table 4).

Exploratory aPL analysis

Among the 48 participants with available aPL results, 23 (47.9%) were aPL-positive, of whom 18 (78.3% of aPL-positive participants) had established organ damage. In the exploratory multivariable model adjusted for SELENA-SLEDAI, aPL positivity was associated with established damage (aOR 5.95, 95% CI 1.44-24.55; *P* = 0.014); higher SELENA-SLEDAI scores were also associated with established damage in this subgroup (aOR 1.12 per point, 95% CI 1.02-1.23; *P* = 0.013) (Table 5). Given the small subgroup size, limited number of outcome events, incomplete aPL testing, and wide confidence interval around the aPL estimate, these findings should be regarded as hypothesis-generating rather than confirmatory.

**Table 5:** Exploratory multivariable analysis of antiphospholipid antibody positivity and organ damage defined by the SLICC/ACR Damage Index. The model included 48 participants with available antiphospholipid antibody testing, 18 damage events, and two predictors, with adjustment for SELENA-SLEDAI. Participants without antiphospholipid antibody testing were not classified as aPL-negative and were therefore excluded from this analysis. *P < 0.05 was considered statistically significant; statistically significant values are denoted by an asterisk. aPL, antiphospholipid antibody; SELENA-SLEDAI, Safety of Estrogens in Lupus National Assessment-Systemic Lupus Erythematosus Disease Activity Index; SLICC/ACR, Systemic Lupus International Collaborating Clinics/American College of Rheumatology; aOR, adjusted odds ratio; CI, confidence interval

Predictor	aOR	95% CI	*P*-value
SELENA-SLEDAI, per point	1.12	1.02-1.23	0.013*
aPL positivity	5.95	1.44-24.55	0.014*

Exploratory medication analysis

Medication exposure was common, particularly to hydroxychloroquine and corticosteroids. None of the five medications examined was significantly associated with established damage in either the exploratory unadjusted or adjusted analyses (Table 6). Hydroxychloroquine was directionally associated with lower odds of established damage, whereas corticosteroids, mycophenolate mofetil, azathioprine, and cyclophosphamide showed directionally higher but imprecise estimates. Because medication exposure was assessed as a binary variable and cumulative dose, duration, adherence, and treatment indication were not adequately captured, these analyses cannot determine whether medication exposure caused or prevented organ damage. The findings should therefore be considered exploratory and may be affected by confounding by indication.

**Table 6:** Exploratory association of medication exposure with organ damage defined by the SLICC/ACR Damage Index. Each medication model was adjusted separately for age, disease duration, SELENA-SLEDAI, and anemia. Medication analyses were exploratory and should not be interpreted as estimates of causal treatment effects, as cumulative medication exposure and treatment indication were incompletely captured. SELENA-SLEDAI, Safety of Estrogens in Lupus National Assessment-Systemic Lupus Erythematosus Disease Activity Index; SLICC/ACR, Systemic Lupus International Collaborating Clinics/American College of Rheumatology; OR, odds ratio; CI, confidence interval

Medication	Exposed, *n* (%)	Unadjusted OR (95% CI)	Adjusted OR (95% CI)	*P*-value (adj.)
Hydroxychloroquine	154 (91.7%)	0.55 (0.18-1.68)	0.92 (0.24-3.53)	0.903
Corticosteroid	125 (74.4%)	1.37 (0.63-2.99)	1.77 (0.72-4.37)	0.214
Mycophenolate mofetil	26 (15.5%)	1.26 (0.52-3.05)	1.49 (0.57-3.90)	0.416
Azathioprine	27 (16.1%)	1.43 (0.61-3.40)	1.18 (0.45-3.12)	0.739
Cyclophosphamide	22 (13.1%)	1.37 (0.54-3.50)	1.27 (0.43-3.72)	0.667

## Discussion

This cross-sectional analysis describes irreversible organ damage among patients with SLE receiving care at a tertiary rheumatology center in Bangladesh. Established SDI-defined damage was present in 51 of 168 patients (30.4%), and most patients with damage had only a modest cumulative burden, with an SDI score of 1 in more than three-quarters of affected patients. Skin damage was the most frequently recorded domain. In the primary adjusted analysis, longer disease duration, anemia, and vasculitis were associated with established damage. Of these, anemia and vasculitis showed the most consistent associations because both remained significant after exclusion of patients with disease duration <1 year.

The observed prevalence of established SDI-defined damage in our study population was 30.4% (51/168). This finding is broadly within the range reported in Asian and Asia-Pacific populations. A recent systematic review of predictors of organ damage in the Asia-Pacific region reported substantial variation in damage prevalence, ranging from approximately 13% to 66% across studies [7]. The prevalence observed in the present study therefore falls within the reported regional spectrum. However, these numerical comparisons should be interpreted cautiously because the included studies differed in disease duration, age distribution, referral setting, clinical phenotype, treatment exposure, sample size, and methods for defining and assessing organ damage. Individual cohorts within this region reported damage in 38% of patients at study entry in southern China [9], 42.9% in a multiethnic Malaysian cohort [11], 47.9% in a retrospective Malaysian cohort [12], and 22.4% at baseline in a large Chinese inception cohort of newly diagnosed patients [16]. The Malaysian cohorts reported longer disease duration (mean 9.2 years [11] and 5.3 years [12]) than our patients (median 3.0 years (IQR 1.0-6.0) without damage and 4.0 years (IQR 2.0-9.0) with damage). Conversely, the lower baseline prevalence in the Chinese inception cohort, which enrolled newly diagnosed patients, is compatible with the short interval since diagnosis in that cohort [16]. Longitudinal APLC data, in contrast, reported damage accrual of approximately 58 per 1000 person-years over a median follow-up of 5.7 years [17]. In contrast, a retrospective Pakistani cohort of 198 patients diagnosed between 1992 and 2005 reported organ damage in 76% (151/198), with renal disease the commonest cause [13]; this exceeds the range summarized in the Asia-Pacific systematic review [7] and may reflect differences in era, follow-up, and case mix. Thus, the 30.4% prevalence observed in our tertiary-care population provides useful local context but should not be interpreted as a population-wide estimate for Bangladesh.

Cutaneous damage was the most frequently recorded damage domain in our study, occurring in 30 patients (17.9% of the total study population and 58.8% of patients with established damage). This pattern differs from some other Asian cohorts in which neuropsychiatric, renal, or musculoskeletal damage has been prominent. For example, a large Korean cohort of 996 patients with SLE reported substantial musculoskeletal, renal, and neuropsychiatric involvement [10]. Malaysian cohorts, in contrast, have described predominantly musculoskeletal and renal damage [11] or neuropsychiatric, skin, and musculoskeletal damage [12]. The predominance of cutaneous damage in our patients may reflect differences in clinical phenotype, disease duration, treatment exposure, referral patterns, and access to specialist care. A Brazilian study also reported that skin damage may occur relatively early and may not be strongly dependent on disease duration [32]. In a Pakistani cohort, renal disease was the commonest cause of damage, whereas alopecia and discoid lesions were univariable predictors of damage [13]; in our patients, by contrast, renal damage was infrequent (1.8%). Data from the multinational APLC cohort also showed that mucocutaneous damage accrued in only 4.3% of patients during a median follow-up of 2.5 years, with alopecia accounting for most of it, although persistent mucocutaneous disease activity increased the risk of such damage and mucocutaneous damage was more frequent in White patients [20]. The much higher frequency of skin damage in our study (17.9%) should therefore be interpreted cautiously because it reflects cumulative damage at a single time point in a tertiary referral population rather than damage accrued during prospective follow-up; ethnic and environmental differences, ascertainment of alopecia, and treatment exposure may also contribute. These findings emphasize that the distribution of irreversible damage may vary substantially between populations even when the overall burden of damage falls within a similar regional range.

The association between disease duration and established damage in the primary model is compatible with the cumulative nature of irreversible injury. Patients have a greater opportunity to experience inflammatory, vascular, thrombotic, or treatment-related events as disease persists, and longitudinal cohorts have demonstrated progressive damage accrual over time [4,5]. In our sensitivity analysis, however, disease duration was no longer significantly associated with established damage after exclusion of patients with disease duration <1 year. Therefore, the primary association should be interpreted cautiously and should not be considered evidence of a simple linear relationship between time since diagnosis and accumulated damage.

Anemia was consistently associated with established damage in both the primary and sensitivity models. Similar observations have been reported in the LUMINA cohort [6]. Anemia in SLE is clinically heterogeneous and may reflect inflammation, iron deficiency, renal dysfunction, hemolysis, medication effects, or other comorbid conditions. In this context, anemia may be better regarded as a marker of overall disease burden or accumulated morbidity rather than as a direct cause of irreversible organ damage. Because of the cross-sectional design, we cannot determine whether anemia preceded established damage or resulted from disease processes associated with damage.

Vasculitis showed a strong and consistent association with established damage in both the primary and sensitivity analyses. This association is biologically plausible because inflammatory vascular injury can result in permanent tissue loss or dysfunction involving cutaneous, neurologic, renal, gastrointestinal, and peripheral vascular tissues. The finding is also consistent with our earlier analysis of lupus-associated vasculitis in the same clinical population, which reported a greater burden of SDI-defined damage among patients with vasculitis [28]. The present study extends that observation by evaluating vasculitis within a broader damage-focused analytical framework. However, because the present analysis is cross-sectional, the temporal relationship between vasculitis and individual damage events cannot be established.

Current SELENA-SLEDAI was associated with established damage in the univariable analysis but was not independently associated with damage after multivariable adjustment. This finding should not be interpreted as evidence that disease activity is unimportant in damage accrual. Rather, SELENA-SLEDAI measures disease activity at the time of assessment, whereas the SDI captures irreversible consequences accumulated over the disease course. A single cross-sectional disease-activity measurement therefore cannot adequately represent cumulative inflammatory exposure. Longitudinal studies have demonstrated associations between sustained disease activity and subsequent damage accrual, while remission and low disease activity have been associated with lower subsequent damage [4,6,25,26]. In the APLC cohort, flares independently increased the risk of damage accrual (adjusted HR 1.32 for mild-to-moderate and 1.58 for severe flares), with a 7% higher risk for each additional flare [17]. Our findings therefore highlight the limitations of using a single current disease-activity score to explain an accumulated outcome.

The exploratory analysis identified an association between aPL positivity and established damage, together with an association between higher SELENA-SLEDAI scores and damage in the 48-patient subgroup. This finding is directionally consistent with previous studies linking aPL to early and overall damage in SLE [21,22]. However, only 48 of the 168 participants had documented aPL results, and the analysis included only 18 damage events. The confidence interval around the aPL estimate was also wide. Furthermore, a single aPL measurement does not establish the persistent antibody positivity required for formal APS classification. Accordingly, these findings should be considered hypothesis-generating rather than confirmatory and should not be extrapolated to the full study population.

The medication analysis did not identify statistically significant associations between hydroxychloroquine, corticosteroids, mycophenolate mofetil, azathioprine, or cyclophosphamide and established damage. These findings should not be interpreted as evidence that these therapies do not influence damage accrual. Medication exposure was assessed as a binary variable, while cumulative dose, duration, adherence, and treatment indication were not consistently available. In particular, standardized information on cumulative glucocorticoid exposure, including prednisolone dose and duration, was insufficient to evaluate the contribution of cumulative glucocorticoid exposure to irreversible damage. This is an important limitation because cumulative glucocorticoid exposure has been associated with damage in longitudinal SLE cohorts [19,23,24]. The absence of standardized cumulative exposure data also leaves the possibility of residual confounding and prevents assessment of dose-response relationships. In addition, patients receiving more intensive treatment may have had more severe disease, creating potential confounding by indication.

The findings have practical implications for SLE care in Bangladesh. Assessment of irreversible damage can provide information that is not captured by a current disease-activity score and may be useful during long-term follow-up. The consistent associations observed with anemia and vasculitis may help clinicians identify patients who warrant careful assessment for established organ injury and potentially modifiable contributors. Preventing future damage remains dependent on sustained disease control while limiting avoidable treatment toxicity, particularly unnecessary long-term glucocorticoid exposure [24,26,27].

Several limitations should be considered. First, the cross-sectional design prevents determination of temporal sequence or causality. Second, the study was conducted at a tertiary rheumatology center, and the sample may therefore include a greater proportion of patients with complex or severe disease than would be observed in the broader Bangladeshi SLE population. Third, this was a secondary analysis of a dataset originally collected for a vasculitis-focused study; the availability, definition, and timing of variables were therefore determined by the original study design, which may introduce selection and information bias, and the overlap with that publication is disclosed [28]. Fourth, disease activity was measured at a single assessment using SELENA-SLEDAI, and cumulative disease activity was unavailable; this may partly explain why current disease activity was not independently associated with damage. Fifth, standardized information on cumulative glucocorticoid exposure, including prednisolone dose and duration, as well as other historical treatment exposures and hypertension, was insufficient for inclusion in the primary model, leaving potential residual confounding and confounding by indication. Sixth, the relatively small sample limited precision for uncommon damage domains and subgroup analyses. In addition, the primary model showed borderline calibration on the Hosmer-Lemeshow test (*P* = 0.053) and only modest discrimination (AUC 0.74 in both models); it was therefore used to describe associations and not to predict damage in individual patients. Seventh, SDI scoring was performed by a single assessor using clinical examination and medical-record information, and formal inter-rater reliability was not assessed. Eighth, definitions of several clinical variables (lupus nephritis, NPSLE, APS, and vasculitis) were applied as in the source dataset and could not be re-evaluated retrospectively, so some misclassification is possible. Ninth, the exploratory aPL analysis (48 patients, 18 damage events) was based on a small, incompletely tested subset, and its estimate is imprecise and may be unstable; it should be considered hypothesis-generating only.

Despite these limitations, this study adds data from an understudied South Asian setting and provides a detailed description of SDI-defined organ damage in a Bangladeshi tertiary-care SLE population. Established damage was present in approximately one-third of participants, while most affected patients had a relatively low SDI burden, and cutaneous damage was the most frequently recorded domain. The consistent associations of anemia and vasculitis with established damage, including after exclusion of patients with very early disease, provide clinically relevant signals that merit evaluation in larger longitudinal Bangladeshi and South Asian cohorts. Future prospective studies incorporating cumulative disease activity, standardized glucocorticoid dose and duration, treatment exposure, and systematic aPL assessment will be important for clarifying the temporal and potentially modifiable determinants of irreversible organ damage.

## Conclusions

Established SDI-defined organ damage was present in approximately one-third of patients with SLE in this Bangladeshi tertiary-care population, with skin involvement representing the most frequently recorded damage domain. Anemia and vasculitis showed the most consistent associations with established damage, whereas the association with disease duration was less robust after excluding patients with very early disease. Current disease activity alone did not fully account for accumulated irreversible damage. These findings support incorporating damage assessment into routine long-term SLE care alongside disease-activity monitoring. Because this was a cross-sectional study, the observed relationships should be interpreted as associations rather than causal effects. Larger prospective studies from Bangladesh and South Asia are needed to clarify how damage develops over time and which factors may be modifiable.

## References

[1] Aringer M, Costenbader K, Daikh D (2019). 2019 European League Against Rheumatism/American College of Rheumatology classification criteria for systemic lupus erythematosus. Arthritis Rheumatol.

[2] Gladman D, Ginzler E, Goldsmith C (1996). The development and initial validation of the Systemic Lupus International Collaborating Clinics/American College of Rheumatology Damage Index for systemic lupus erythematosus. Arthritis Rheum.

[3] Murimi-Worstell IB, Lin DH, Nab H (2020). Association between organ damage and mortality in systemic lupus erythematosus: a systematic review and meta-analysis. BMJ Open.

[4] Bruce IN, O'Keeffe AG, Farewell V (2015). Factors associated with damage accrual in patients with systemic lupus erythematosus: results from the Systemic Lupus International Collaborating Clinics inception cohort. Ann Rheum Dis.

[5] Petri M, Purvey S, Fang H, Magder LS (2012). Predictors of organ damage in systemic lupus erythematosus: the Hopkins Lupus Cohort. Arthritis Rheum.

[6] Bertoli AM, Vilá LM, Apte M, Fessler BJ, Bastian HM, Reveille JD, Alarcón GS (2007). Systemic lupus erythematosus in a multiethnic US cohort LUMINA LI: anaemia as a predictor of disease activity and damage accrual. Rheumatology (Oxford).

[7] Ramnarain A, Liam C, Milea D, Morand E, Kent J, Kandane-Rathnayake R (2024). Predictors of organ damage in systemic lupus erythematosus in the Asia Pacific region: a systematic review. Arthritis Care Res (Hoboken).

[8] Thumboo J, Wee HL (2006). Systemic lupus erythematosus in Asia: is it more common and more severe?. APLAR J Rheumatol.

[9] Mok CC, Ho CTK, Wong RWS, Lau CS (2003). Damage accrual in southern Chinese patients with systemic lupus erythematosus. J Rheumatol.

[10] Joo YB, Bae SC (2015). Assessment of clinical manifestations, disease activity and organ damage in 996 Korean patients with systemic lupus erythematosus: comparison with other Asian populations. Int J Rheum Dis.

[11] Shaharir SS, Hussein H, Rajalingham S, Mohamed Said MS, Abdul Gafor AH, Mohd R, Mustafar R (2016). Damage in the multiethnic Malaysian systemic lupus erythematosus cohort: comparison with other cohorts worldwide. PLoS One.

[12] Ghazali WS, Daud SM, Mohammad N, Wong KK (2018). Slicc damage index score in systemic lupus erythematosus patients and its associated factors. Medicine (Baltimore).

[13] Rabbani MA, Habib HB, Islam M (2009). Survival analysis and prognostic indicators of systemic lupus erythematosus in Pakistani patients. Lupus.

[14] Rabbani MA, Habib HB, Islam M (2010). Early renal damage assessed by the SLICC/ACR damage index is predictor of severe outcome in lupus patients in Pakistan. Lupus.

[15] Wada Y, Hasegawa H, Saeki T, Ito S, Kuroda T, Nakano M, Narita I (2018). Long-term prognosis and factors associated with damage accrual in Japanese patients with systemic lupus erythematosus. Clin Exp Nephrol.

[16] Gui Y, Bai W, Xu J (2022). Sex differences in systemic lupus erythematosus (SLE): an inception cohort of the Chinese SLE Treatment and Research Group (CSTAR) registry XVII. Chin Med J (Engl).

[17] Kandane-Rathnayake R, Milea D, Louthrenoo W (2025). Longitudinal associations of flare and damage accrual in patients with systemic lupus erythematosus. Lupus Sci Med.

[18] Sunny SS, Islam MI, Bandhan IH (2026). Gender-based differences in clinical, immunological and disease activity profiles of systemic lupus erythematosus patients in Bangladesh. Mymensingh Med J.

[19] Kandane-Rathnayake R, Kent JR, Louthrenoo W (2019). Longitudinal associations of active renal disease with irreversible organ damage accrual in systemic lupus erythematosus. Lupus.

[20] Saracino AM, Hao Y, Hansen D (2026). Mucocutaneous disease activity and damage accrual in systemic lupus erythematosus: analyses from the Asia-Pacific Lupus Collaboration longitudinal cohort study. Arthritis Care Res (Hoboken).

[21] Ruiz-Irastorza G, Egurbide MV, Martinez-Berriotxoa A, Ugalde J, Aguirre C (2004). Antiphospholipid antibodies predict early damage in patients with systemic lupus erythematosus. Lupus.

[22] Taraborelli M, Leuenberger L, Lazzaroni MG (2016). The contribution of antiphospholipid antibodies to organ damage in systemic lupus erythematosus. Lupus.

[23] Fessler BJ, Alarcón GS, McGwin G Jr (2005). Systemic lupus erythematosus in three ethnic groups: XVI. Association of hydroxychloroquine use with reduced risk of damage accrual. Arthritis Rheum.

[24] Apostolopoulos D, Kandane-Rathnayake R, Raghunath S, Hoi A, Nikpour M, Morand EF (2016). Independent association of glucocorticoids with damage accrual in SLE. Lupus Sci Med.

[25] Apostolopoulos D, Kandane-Rathnayake R, Louthrenoo W (2020). Factors associated with damage accrual in SLE with no clinical or serological disease activity: multicentre cohort. Lancet Rheumatol.

[26] Ugarte-Gil MF, Hanly J, Urowitz M (2022). Remission and low disease activity (LDA) prevent damage accrual in patients with systemic lupus erythematosus: results from the Systemic Lupus International Collaborating Clinics (SLICC) inception cohort. Ann Rheum Dis.

[27] Kandane-Rathnayake R, Zolio L, Choi JI (2026). Predictors of damage accrual by organ domain in systemic lupus erythematosus. Rheumatology (Oxford).

[28] Sunny SS, Azad MA, Hassan MM, Asaduzzaman A, Islam MN (2025). Predictors of vasculitis in patients with systemic lupus erythematosus in Bangladesh. Cureus.

[29] Bombardier C, Gladman DD, Urowitz MB (1992). Derivation of the SLEDAI. A disease activity index for lupus patients. Arthritis Rheum.

[30] Petri M, Kim MY, Kalunian KC (2005). Combined oral contraceptives in women with systemic lupus erythematosus. N Engl J Med.

[31] von Elm E, Altman DG, Egger M, Pocock SJ, Gøtzsche PC, Vandenbroucke JP (2007). The Strengthening the Reporting of Observational Studies in Epidemiology (STROBE) statement: guidelines for reporting observational studies. PLoS Med.

[32] Guarize J, Appenzeller S, Costallat LT (2007). Skin damage occurs early in systemic lupus erythematosus and independently of disease duration in Brazilian patients. Rheumatol Int.

